# Bayesian Method with Spatial Constraint for Retinal Vessel Segmentation

**DOI:** 10.1155/2013/401413

**Published:** 2013-07-14

**Authors:** Zhiyong Xiao, Mouloud Adel, Salah Bourennane

**Affiliations:** Institut Fresnel/UMR-CNRS, D. U. de Saint-Jérôme, 13013 Marseille, France

## Abstract

A Bayesian method with spatial constraint is proposed for vessel segmentation in retinal images. The proposed model makes the assumption that the posterior probability of each pixel is dependent on posterior probabilities of their neighboring pixels. An energy function is defined for the proposed model. By applying the modified level set approach to minimize the proposed energy function, we can identify blood vessels in the retinal image. Evaluation of the developed method is done on real retinal images which are from the DRIVE database and the STARE database. The performance is analyzed and compared to other published methods using a number of measures which include accuracy, sensitivity, and specificity. The proposed approach is proved to be effective on these two databases. The average accuracy, sensitivity, and specificity on the DRIVE database are 0.9529, 0.7513, and 0.9792, respectively, and for the STARE database 0.9476, 0.7147, and 0.9735, respectively. The performance is better than that of other vessel segmentation methods.

## 1. Introduction

 Retinal vessel segmentation plays an important role in medical image processing. It can provide much help for the detection of eye diseases and other medical diagnosis. A large number of methods for retinal vessel segmentation have been proposed. A survey on retinal vessel segmentation methods is presented in the literature [1]. According to the image processing methodologies and algorithms, these retinal vessel segmentation approaches can be categorized into pattern recognition techniques, matched filtering, vessel tracking, mathematical morphology, multiscale approaches, and model-based approaches.

The algorithm based on pattern recognition can detect or classify the retinal blood vessel features and the background. This group of algorithms can be divided into two categories: supervised and unsupervised approaches. In supervised methods, the prior labeling information is used to decide whether a pixel belongs to a vessel or not. In [2], a ridge-based vessel segmentation methodology has been proposed. In [3], a method which combines the radial projection and the support vector machines classifier has been introduced for vessel segmentation. A supervised method which is based on neural network has been presented in [4]. The Gaussian matched filter and the k-nearest neighbor algorithm are used for vessel segmentation [5]. In [6], a 2D Gabor wavelet has been applied for vessel segmentation. The unsupervised methods perform the vessel segmentation without any prior labeling knowledge. In [7], a spatially weighted fuzzy C-means clustering method has been used for vessel segmentation. In [8], a vessel detection system based on a maximum likelihood estimation has been developed.

The matched filtering approaches [9–11] are also popular methods to detect and measure blood vessels. In [9], a method which combines local and region-based properties of retinal blood vessels has been described. In [10], a modified second-order Gaussian filter has been used for retinal vessel detection. In [11], the zero-mean Gaussian filter and the first-order derivative of the Gaussian have been applied to detect vessels.

In the vessel tracking-based method framework [12–14], several seed pixels are chosen on the boundaries and the centerlines of vessels, and then we detect vessels from these seeded pixels. In [13], the Bayesian method with the maximum a posteriori (MAP) probability criterion has been used to identify the vessel's boundary points. In [12, 14], a probabilistic tracking method has been used to detect the vessel edge points by using local grey level statistics and vessel's continuity properties.

The mathematical morphology-based methods extract image components which are useful in the representation and description of region shapes such as features, boundaries, skeletons, and convex hulls. In [15], a unique combination of vessel centerlines detection and morphological bit plane slicing has been introduced to extract the blood vessel tree form the retinal images. The fast discrete curvelet transforms and multistructure mathematical morphology have been employed for vessel detection [16]. In [17], the combination of morphological filters and cross-curvature evaluation have been used to segment vessel-like patterns. A difference of offset gaussian filter [18] has been utilized for retinal vasculature extraction. A general framework of locally adaptive thresholding method for retinal vessel segmentation has been introduced in [19].

Multiscale approaches are to separate out information related to the blood vessel having varying width at different scales. A scale space segmentation algorithm has been proposed in [20], which has been used to measure and quantify geometrical and topological properties of the retinal vascular tree. Two extensions of this scale space algorithm have been demonstrated in [21, 22]. A multi-scale line tracking for vasculature segmentation has been presented in [23].

The model-based approaches are very popular techniques for image segmentation and have been used for retinal vessel segmentation. Active contour models which are based on curve evolution are very commonly used for image segmentation. The main advantage is their great performance. Recently, they have been used to detect boundaries of vessels in retinal images [24–28]. The classical snake in combination with blood vessel topological properties has been used to extract the vasculature from retinal image [29]. A methodology based on nonlinear projections has been proposed for vessel segmentation [30]. Level set method is a very good approach to deal with topological changes [31] and has been successfully used for vessel segmentation of retinal images [32, 33]. Graph-based approach is very popular and interesting method for image segmentation and also has been applied to vessel boundary detection [34].

The above methods [12–14] are based on the Bayesian model. However, the main disadvantage of these approaches is that the pixels are assumed to be independent. Spatial dependence is very important to guarantee connectedness of the vessel structure. To take into account the dependence in the spatial space, Markov random field (MRF) models have been widely used for solving the image segmentation problem [35–37]. However, one of the drawbacks of the MRF-based methods is that the computational cost is quite high.

In this paper, we present a novel Bayesian segmentation method for vessel segmentation. The proposed method takes the spatial information into account. We found that maximizing log-likelihood function is equivalent to energy function minimization. The parameters of the model can be estimated via energy minimization. In order to detect the boundaries of the blood vessels, the modified level set approach is used for solving the energy function minimization problem. The method was evaluated on two publicly available databases, the DRIVE database [38] and the STARE database [39]. Results of the proposed method are compared to those from other methods, leading to the conclusion that our approach outperforms other techniques.

The remainder of this paper is organized as follows. In Sections 2 and 3, we describe the details of the proposed model and the modified level set algorithm. In Section 4, we show the experimental results and conclude with a discussion in Section 5.

## 2. Proposed Model

### 2.1. Image Segmentation

Let *𝒳* = {*x*_*i*_, *i* ∈ *Ω*} denote an observed image, where *x*_*i*_ is the observation of pixel *i* and *Ω* is image domain. Let *𝒦* = {1,2,…, *K*} denote a label set, and *K* is the total number of classes. Let *𝒴* = {*y*_*i*_ ∈ *𝒦*, *i* ∈ *Ω*} be an image of labels. The aim of labeling is to assign a label *y*_*i*_ ∈ *𝒦* to each pixel *i* ∈ *Ω*, based on *x*_*i*_. The goal of segmentation is to separate the image domain *Ω* into disjoint regions *Ω*_1_,…, *Ω*_*K*_ and ensure smooth inside each region *Ω*_*k*_. Notice that, given a labeling *𝒴*, the collection *Ω*_*k*_ = {*i* ∈ *Ω* | *y*_*i*_ = *k*} for *k* ∈ *𝒦* is one of these regions. Also, given the segmentation *Ω*_*k*_ for *k* ∈ *𝒦*, the image {*y*_*i*_ | *y*_*i*_ = *k*  if  *i* ∈ *Ω*_*k*_, *i* ∈ *Ω*} is a labeling. It is a one-to-one relationship between the labeling and the segmentation. Thus, the image segmentation problem can be considered as a labeling problem.

### 2.2. Bayesian Model with Spatial Constraint

In Bayesian framework, inference is often carried out by maximizing the posterior distribution

(1) $$\begin{array}{c} P\left(𝒴\;|\;𝒳\right)\propto P\left(𝒳\;|\;𝒴\right)P\left(𝒴\right), \end{array}$$

where *P*(*𝒳* | *𝒴*) is the likelihood function and *P*(*𝒴*) is the prior distribution.

When the pixels are considered independent of each other, the likelihood function can be written as

(2) P(𝒳 ∣ 𝒴)=∏i∈ΩP(xi ∣ yi)=∏k=1K ∏i∈ΩkP(xi ∣ yi=k).

However, the spatial relationships between neighboring pixels are not taken into account. To improve the accuracy of the segmented results, the spatial dependencies should be taken into account.

In this paper, we use a modeling strategy for the spatial dependencies between the conditional probabilities. The conditional probability *P*(*x*_*i*_ | *y*_*i*_) is defined as a mixture distribution over the conditional probabilities of neighboring pixels *j*, *j* ∈ *𝒩*_*i*_, that is,

(3) $$\begin{array}{c} P\left(x_{i}\;|\;y_{i}\right)=\prod_{j\in {𝒩}_{i}}\lambda_{ij}P\left(x_{j}\;|\;y_{j}\right), \end{array}$$

where *λ*_*ij*_ are fixed positive weights and for each *i* holds ∑_*j*_ *λ*_*ij*_ = 1. The mixing weight *λ*_*ij*_ depends on the geometric closeness between the pixels *i* and *j*. Thus, the above likelihood function can be expressed as

(4) P(𝒳 ∣ 𝒴)=∏i∈Ω∏j∈𝒩iλijP(xj ∣ yj)=∏i∈Ω ∏k=1K∏j∈𝒩i⋂ΩkλijP(xj ∣ yj=k).

Let *P*(*x*_*i*_ | *θ*_*k*_) be the parameter form of the *P*(*x*_*i*_ | *y*_*i*_ = *k*). Usually, it is assumed to be Gaussian distribution

(5) $$\begin{array}{c} P\left(x_{i}\;|\;\theta_{k}\right)=\frac{1}{\sqrt{2\pi \sigma_{k}^{2}}}\exp\left(-\frac{{\left(x_{i}-\mu_{k}\right)}^{2}}{2\sigma_{k}^{2}}\right). \end{array}$$

The log-likelihood function of Bayesian model (4) is given by

(6) ℒ(μ,σ) =∑i∈Ω ∑k=1K∑j∈𝒩i⋂Ωklog⁡(λijP(xj ∣ θk))  =∑i∈Ω ∑k=1K∑j∈𝒩i⋂Ωk[log⁡λij+log⁡P(xj ∣ θk)]  =∑i∈Ω ∑k=1K∑j∈𝒩i⋂Ωk[log⁡λij−12log⁡(2πσk2)−(xj−μk)22σk2],

where *λ*_*ij*_ are fixed positive weights and for each *i* it holds ∑_*j*_ *λ*_*ij*_ = 1. The mixing weight *λ*_*ij*_ depends on the geometric closeness between the pixels *i* and *j* [40].

The geometric closeness *h*_*ij*_ is a Gaussian function of the magnitude of the relative position vector of pixel *j* from pixel *i*, ||*u*_*i*_ − *u*_*j*_||. The geometric closeness function is given as a decreasing function when the distance ||*u*_*i*_ − *u*_*j*_|| increases as

(7) $$\begin{array}{c} h_{ij}=\exp\left(\frac{-{||u_{i}-u_{j}||}^{2}}{2\sigma_{g}^{2}}\right), \end{array}$$

where *σ*_*g*_ is parameter, which defines the desired structural locality between neighboring pixels, and *u*_*i*_ and *u*_*j*_ are the location of the pixel *i* and *j*, respectively. We use a 3 × 3 neighborhood window and we suggest *σ*_*g*_^2^ = 10.

We define the mixing weight *λ*_*ij*_ as follows:

(8) $$\begin{array}{c} \lambda_{ij}=\frac{h_{ij}}{\sum_{j\in N(i)}^{}h_{ij}}. \end{array}$$

Note that *λ*_*ij*_ are fixed constants, and we can drop the term that depends only on *λ*_*ij*_. Image segmentation can be performed by maximizing log-likelihood function *ℒ*(*μ*, *σ*) (6) with respect to the parameter *μ*, *σ* as

(9) argmax⁡ μ,σℒ(μ,σ) ⇔argmax⁡μ,σ−12∑i∈Ω ∑k=1K∑j∈𝒩i⋂Ωk[log⁡(2πσk2)                     +(xj−μk)2σk2].

### 2.3. Energy Minimization

Since the logarithm is a monotonically increasing function, it it more convenient to consider the negative likelihood function as an energy function as

(10) $$\begin{array}{c} ℰ\left(\mu ,\sigma \right)=\frac{1}{2}\sum_{i\in \Omega }\;\sum_{k=1}^{K}\sum_{j\in {𝒩}_{i}⋂\Omega_{k}}\left[\log\left(2\pi \sigma_{k}^{2}\right)+\frac{{\left(x_{j}-\mu_{k}\right)}^{2}}{\sigma_{k}^{2}}\right]. \end{array}$$

Thus, image segmentation problem can be solved by minimizing energy *ℰ*(*μ*, *σ*) (10) with respect to the parameter *μ*, *σ*.

We assume that the variance of the proposed energy (10) has the common form *σ*. Thus, the functional for the proposed energy (10) can be written as

(11) $$\begin{array}{c} ℱ\left(\mu \right)=\sum_{i\in \Omega }\;\sum_{k=1}^{K}\sum_{j\in {𝒩}_{i}⋂\Omega_{k}}{\left(x_{j}-\mu_{k}\right)}^{2}. \end{array}$$

We introduce kernel function *ρ*(*x*_*i*_, *x*_*j*_) as a nonnegative window function

(12) $$\begin{array}{c} \rho \left(x_{i},x_{j}\right)=\{\begin{array}{cc} 1, & \text{if}\;\;j\in {𝒩}_{i},\\ 0, & \text{if}\;\;\text{otherwise}. \end{array} \end{array}$$

With the window function, the energy function (11) can be rewritten as

(13) $$\begin{array}{c} ℱ\left(\mu \right)=\sum_{i\in \Omega }\;\sum_{k=1}^{K}\sum_{j\in \Omega_{k}}\rho \left(x_{i},x_{j}\right){\left(x_{j}-\mu_{k}\right)}^{2}. \end{array}$$

By exchanging the order of sum, we have

(14) $$\begin{array}{c} ℱ\left(\mu \right)=\sum_{k=1}^{K}\;\sum_{j\in \Omega_{k}}\sum_{i\in \Omega }\rho \left(x_{i},x_{j}\right){\left(x_{j}-\mu_{k}\right)}^{2}. \end{array}$$

For convenience, we can rewrite the above energy functional *ℱ*(*μ*) in the following form:

(15) $$\begin{array}{c} ℱ\left(\mu \right)=\sum_{k=1}^{K}\;\sum_{j\in \Omega_{k}}e_{k}\left(x_{j}\right), \end{array}$$

where *e*_*k*_(*x*_*j*_) is the function defined by

(16) $$\begin{array}{c} e_{k}\left(x_{j}\right)=\sum_{i\in \Omega }\rho \left(x_{i},x_{j}\right){\left(x_{j}-\mu_{k}\right)}^{2}. \end{array}$$

We minimize the above proposed energy functional *ℱ*(*μ*) using the modified level set approach.

## 3. Algorithm

In this section, the energy (10) is converted to a level set formulation by representing the disjoint regions *Ω*_1_,…, *Ω*_*K*_ with a number of level set functions, with a regularization term on these level set functions.

We consider the two-phase level set formulation. The image domain *𝒳* is segmented into two disjoint regions *Ω*_1_ and *Ω*_2_. In level set methods, a level set function is a function that takes positive and negative signs. We use level set function to represent a partition of the domain *𝒳* into two disjoint regions *Ω*_1_ and *Ω*_2_ as

(17) $$\begin{array}{c} \Omega_{1}=\left\{x_{i},\;\phi \left(x_{i}\right)>0\right\},\;\;\Omega_{2}=\left\{x_{i},\;\phi \left(x_{i}\right)<0\right\}. \end{array}$$

The regions *Ω*_1_ and *Ω*_2_ can be represented with their membership functions defined by the Heaviside function *H*(*ϕ*) as

(18) $$\begin{array}{c} H\left(\phi \right)=\{\begin{array}{cc} 1, & \text{if}\;\;\phi \geq 0,\\ 0, & \text{if}\;\;\phi <0, \end{array}\\ \delta \left(\phi \right)=\frac{d}{d\phi }H\left(\phi \right). \end{array}$$

Thus, the energy (15) can be expressed as the following level set formulation:

(19) ℱ(μ)=∑j∈Ω1e1(xj)H(ϕ(xj))+∑j∈Ω2e2(xj)(1−H(ϕ(xj))),

where *e*_*k*_(*x*_*j*_) is defined in (16). The level set function *ϕ* and the parameters *μ* are the variables of the energy *ℱ* (19).

Let *ℒ*(*ϕ*) and *ℛ*(*ϕ*) denote the regularization terms of level set function *ϕ*. The energy term *ℒ*(*ϕ*) is defined by [41]

(20) ℒ(ϕ)=∑i∈Ω|∇H(ϕ(xi))|=∑i∈Ωδ(ϕ(xi))|∇ϕ(xi)|,

which computes the arc length of the zero level contour of *ϕ* and serves to smooth the contour [41]. The energy term *ℛ*(*ϕ*) is defined by [42]

(21) $$\begin{array}{c} ℛ\left(\phi \right)=\sum_{i\in \Omega }\frac{1}{2}{\left(\left|\nabla \phi \left(x_{i}\right)\right|-1\right)}^{2}, \end{array}$$

where function *ℛ* is an energy density function, which is called a distance regularization term [42].

Therefore, combining these two energy terms with the energy *ℱ* (19), the total energy functional becomes

(22) ℱ(ϕ,μ)=ℱ(ϕ,μ)+αℒ(ϕ)+βℛ(ϕ)=∑j∈Ω1e1(xj)H(ϕ(xj))+∑j∈Ω2e2(xj)(1−H(ϕ(xj)))+α∑i∈Ωδ(ϕ(xi))|∇ϕ(xi)|+β∑i∈Ω12(|∇ϕ(xi)|−1)2.

By minimizing the above energy, we obtain the result of image segmentation given by the level set function *ϕ* and the estimation of the parameters *μ*. The details of the algorithm for minimizing the energy (22) are given in the next section.

### 3.1. Modified Level Set Algorithm

The energy minimization is achieved by an iterative process: in each iteration, we minimize the energy *ℱ*(*ϕ*, *μ*) (22) with respect to each of its variables *ϕ*, *μ*, given the other two updated in previous iteration. We give the solution to the energy minimization with respect to each variable as follows.

Keeping *μ* fixed and minimizing *ℱ*(*ϕ*, *μ*) (22) with respect to *ϕ*, we use the gradient descent method to solve the gradient flow equation as

(23) $$\begin{array}{c} \frac{\partial \phi }{\partial t}=-\frac{\partial ℱ}{\partial \phi }, \end{array}$$

where ∂*ℱ*/∂*ϕ* is the Gâteaux derivative [41] of the energy *ℱ*.

We compute the Gâteaux derivative ∂*ℱ*/∂*ϕ* and the corresponding gradient flow equation is

(24) ∂ϕ∂t=δ(ϕ)(e2−e1)+αδ(ϕ)div⁡(∇ϕ|∇ϕ|)+βdiv⁡((1−1|∇ϕ|)∇ϕ).

During the evolution of the level set function according to (24), the variables *μ* are updated by minimizing the energy *ℱ*(*ϕ*, *μ*) (22) with respect to *μ*.

Keeping *ϕ* fixed and minimizing *ℱ*(*ϕ*, *μ*) (22) with respect to *μ*, the variable *μ* can be expressed as

(25) $$\begin{array}{c} \mu_{1}=\frac{\sum_{i\in \Omega }\sum_{j\in {𝒩}_{i}}x_{j}H\left(\phi \left(x_{j}\right)\right)‍\;‍}{\sum_{i\in \Omega }\sum_{j\in {𝒩}_{i}}H\left(\phi \left(x_{j}\right)\right)},\\ \mu_{2}=\frac{\sum_{i\in \Omega }\sum_{j\in {𝒩}_{i}}x_{j}\left(1-H\left(\phi \left(x_{j}\right)\right)\right)}{\sum_{i\in \Omega }\sum_{j\in {𝒩}_{i}}\left(1-H\left(\phi \left(x_{j}\right)\right)\right)}. \end{array}$$

Finally, the principal steps of the algorithm are as follows.

- Initialize *ϕ* ^*n*^, *n* = 0.
- Compute *μ*_*k*_^*n*^, *k* = 1,2 by (25).
- Solve the PDE ∂*ϕ*/∂*t* = 0 where ∂*ϕ*/∂*t* is defined in (24) to obtain *ϕ* ^*n*+1^.
- Check whether the solution is stationary. If not, let *n* = *n* + 1 and repeat.

### 3.2. Numerical Approximation

In numerical implementation, in order to compute the unknown function *ϕ*, we consider the slightly regularized version of the Heaviside function *H*, denoted here by *H*_*ε*_, which is computed by [41]

(26) $$\begin{array}{c} H_{\varepsilon }\left(z\right)=\frac{1}{2}\left[1+\frac{2}{\pi }\arctan\left(\frac{z}{\varepsilon }\right)\right]. \end{array}$$

Accordingly, the dirac delta function *δ*, which is the derivative of the Heaviside function, is replaced by the derivative of approximation Heaviside function *H*_*ε*_. The dirac delta function *δ* is given by

(27) $$\begin{array}{c} \delta_{\varepsilon }\left(z\right)=\frac{1}{\pi }\frac{\varepsilon }{\varepsilon^{2}+z^{2}}. \end{array}$$

Since the energy is nonconvex, the solution may be the local minima. With the Heaviside function *H*_*ε*_ and the dirac delta function *δ*_*ε*_, the algorithm has the tendency to compute the global minimizer. Thus, the algorithm is not sensitive to the position of the initial curve.

## 4. Experiments

 In this section, we have evaluated and compared the proposed method for the segmentation of retinal images. The retinal images are obtained from two publicly available databases the DRIVE database [38] and the STARE database [39]. In the experiments, we generally choose the parameters as follows: *α* = 1.0 and the time step Δ*t* = 0.1. After many experiments on a small number of example images, we have found that, when *β* = 0.001 × 255^2^, the performance is very good. In all the following experiments, the values of the parameters are same.

The images of the DRIVE database are with 565 × 584 pixels and 8 bits per color channel. The database includes binary images with the results of manual segmentation, which have been used as ground truth to evaluate the performance of the vessel segmentation methods. The retinal images of the STARE database are digitized to 700 × 605 pixels, 8 bits per RGB channel. The STARE database contains 20 images for blood vessel segmentation: 10 normal images and 10 abnormal images. Binary images with manual segmentations are also available for each image of this database.

Evaluation of the developed method is done on the DRIVE and STARE databases. Experimental results are compared to those obtained using other vessel segmentation methods. To facilitate the comparison with other retinal vessel segmentation methods, the segmentation accuracy has been selected as performance measure. The segmentation accuracy has been defined by the ratio of the total number of correctly classified pixels by the number of pixels in the field of view (FOV). It contains values in the range [0,1], with values closer to 1 indicating a good result. Other important measures are sensitivity and specificity. In this paper, the sensitivity is estimated by the percentage of pixels correctly classified as vessel pixels. The specificity stands for the fraction of pixels erroneously classified as vessel pixels. The ground truth for evaluating the performance measures was a manual segmentation result which is provided together with each database image.

A majority of the pixels are often easy to be classified by the previous methods; however, some of the pixels, such as those on the boundary of a vessel, those for small vessels, and those for vessels near pathology, are difficult to be classified. The proposed method provides a novel way to account for spatial dependence between image pixels. Thus, it can reduce the sensitivity of the segmented results and guarantee connectedness of the vessel structure.

**Figure 1:**
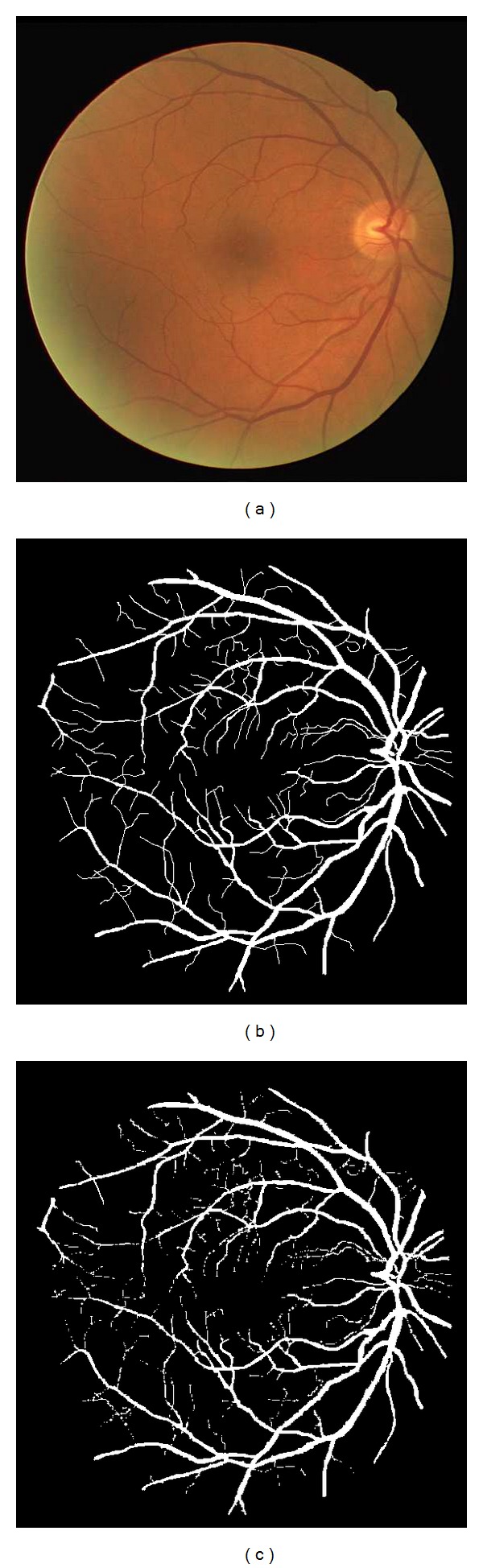
Experiment on two retinal images of DRIVE database. (a) Original retinal images. (b) Ground truth. (c) Segmentation results obtained by the proposed method.

In the first experiment, the proposed method is done on retinal vessel images of DRIVE database. Image in Figure 1(a) shows the original retinal vessel images. The ground truth of the original retinal image is presented in Figure 1(b). The segmentation result obtained by using the proposed method is illustrated in Figure 1(c). It can observed that the proposed method obtains good results.

**Figure 2:**
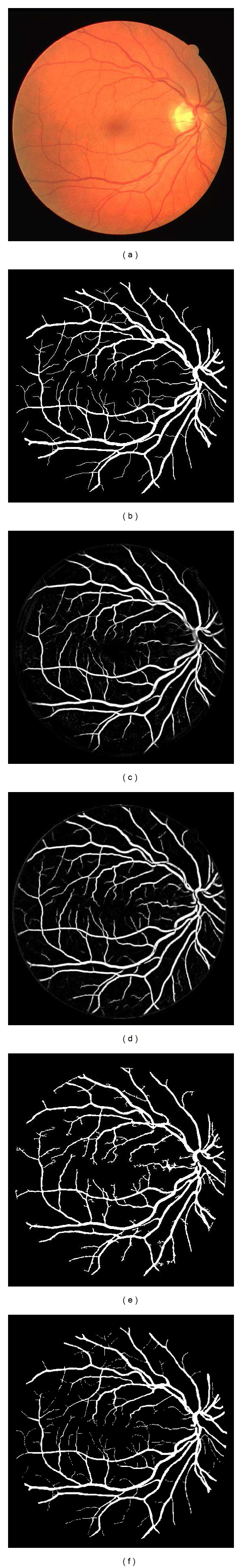
Experiment on retinal image of DRIVE database. (a) Original retinal image. (b) Ground truth. (c)–(f) Segmentation result obtained by Niemeijer et al.'s method [5], Staal et al.'s method [2], Mendonça and Campilho (green intensity) method [18], and the proposed method, respectively.

In order to test the accuracy and determine the efficiency of the proposed method, we do experiment on another retinal vessel image of the DRIVE database and compare the result with those obtained by other methods.  Figure 2(a) shows a retinal image of the DRIVE database. The image in Figure 2(b) is the ground truth. Figures 2(c)–2(f) present the segmentation results obtained by Niemeijer et al.'s method [5], Staal et al.'s method [2], Mendonça and Campilho (green intensity) method [18], and the proposed method, respectively. For visual inspection of the results, the proposed method produces a very good segmentation result.

To facilitate the comparison of our results to those presented by other authors in their original papers, the results have been calculated for the images of the DRIVE database. The value results of the proposed method are shown in Table 1. The other vessel segmentation methods which are reported in their published papers are also presented in Table 1. The performance measures of the proposed method in Table 1 are the average values for all the images of DRIVE database. We can view that the proposed method can capture more correctly classified vessel pixels and less erroneously classified vessel pixels than the other methods. The average accuracy of the proposed method is better than the other techniques.

**Figure 3:**
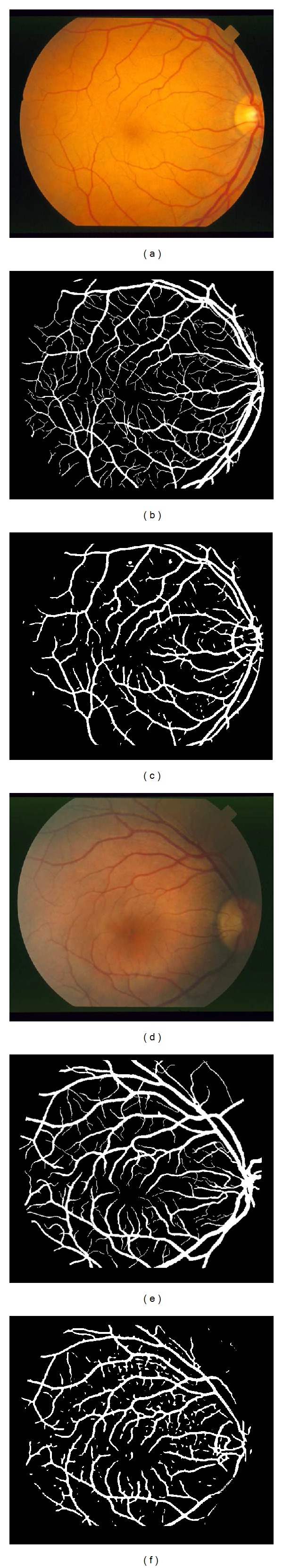
Experiment on retinal images of STARE database. (a), (d) Original retinal images. (b), (e) Ground truth. (c), (f) Segmentation results obtained by the proposed method.

In order to further test the accuracy and determine the efficiency of the proposed method, the proposed method has been tested on the images of the STARE database. Figures 3(a) and 3(d) show two retinal images of STARE database. Figures 3(b) and 3(e) present the ground truth, respectively. The results obtained by using the proposed method are shown in Figures 3(c) and 3(f), respectively. For visual inspection, it can be seen that the proposed method can obtain very good results.

To compare our results to those reported in other published papers, we give the performance measures in Table 2. The average accuracy, sensitivity, and specificity are used to measure the performance. We can note that the proposed method performs better than the other techniques.

The execution time of the proposed method depends on many parameters. For the images of the STARE database, one iteration time of the proposed method is less than 10 seconds. Convergence of the proposed algorithm may be achieved in less than 10 iterations.

## 5. Conclusion

 The accurate extraction of the retinal blood vessel can provide much help for diagnosis of cardiovascular and ophthalmologic diseases. Even though many techniques and algorithms have been developed, there is still room for improvement in blood vessel segmentation methodologies. In this paper, we have presented a novel Bayesian model for vessel segmentation. To overcome the drawback that the spatial information is not taken into account, the proposed model exploits the spatial information. To obtain the boundaries of the blood vessels, the modified level set approach is employed for minimizing the proposed energy function. The proposed method has been tested on real retinal image databases and the experimental results have been compared with those of other methods. Since the developed method takes the spatial information into account, it can result in very good performance in the detection of narrow and low contrast vessels and guarantee connectedness of the vessel structure. The comparison demonstrates that the proposed method outperforms other methods. In future work, we will compare the proposed method to other algorithms. Most of the techniques in the literature and the proposed method are evaluated on a limited range of databases (DRIVE and STARE). We will do more experiments on larger database and compare to more techniques in future.

## Figures and Tables

**Table 1:** Performance of vessel segmentation methods (DRIVE images).

Method	Average accuracy	Sensitivity	Specificity
Staal et al. [2]	0.9442	0.7194	0.9773
You et al. [3]	0.9434	0.7410	0.9751
Marín et al. [4]	0.9452	0.7067	0.9801
Niemeijer et al. [5]	0.9417	0.6898	0.9696
Zhang et al. [11]	0.9382	0.7120	0.9724
Yin et al. [12]	0.9267	0.6252	0.9710
Fraz et al. [15]	0.9430	0.7152	0.9769
Miri and Mahloojifar [16]	0.9458	0.7352	0.9795
Mendonça and Campilho [18]	0.9452	0.7344	0.9764
Martinez-Perez et al. [21]	0.9344	0.7246	0.9655
Martinez-Perez et al. [22]	0.9220	0.6602	0.9612
Vlachos and Dermatas [23]	0.9291	0.7472	0.9550
Espona et al. [29]	0.9352	0.7436	0.9615
Proposed Method	0.9529	0.7513	0.9792

**Table 2:** Performance of vessel segmentation methods (STARE images).

Method	Average accuracy	Sensitivity	Specificity
Staal et al. [2]	0.9442	0.7194	0.9773
Soares et al. [6]	0.9454	0.7212	0.9730
Hoover et al. [9]	0.9267	0.6751	0.9567
Yin et al. [12]	0.9413	0.7249	0.9666
Fraz et al. [15]	0.9442	0.7311	0.9680
Mendonça and Campilho [18]	0.9440	0.6996	0.9730
Martinez-Perez et al. [21]	0.9410	0.7506	0.9569
Martinez-Perez et al. [22]	0.9240	0.7790	0.9409
Zhang et al. [30]	0.9087	0.7373	0.9736
Proposed Method	0.9476	0.7147	0.9735
